# Occult senile cardiac amyloid in severe calcific aortic stenosis is not rare and has a poor prognosis: a 146 patient CMR biopsy study

**DOI:** 10.1186/1532-429X-18-S1-O40

**Published:** 2016-01-27

**Authors:** Thomas A Treibel, Marianna Fontana, Janet A Gilbertson, Karen A Boniface, Steven K White, Amna Abdel-Gadir, Stefania Rosmini, David F Hutt, Carol J Whelan, Julian D Gillmore, Ashutosh Wechalekar, Martin P Hayward, Michael A Ashworth, Philip N Hawkins, James C Moon

**Affiliations:** 1 Cardiac Imaging, Barts Heart Centre, London, UK; 2 grid.83440.3b0000000121901201Institute of Cardiovascular Sciences, University College London, London, UK; 3 grid.83440.3b0000000121901201National Amyloidosis Centre, Royal Free Campus, University College London, London, UK; 4 grid.420468.cDepartment of Histopathology, Great Ormond Street Hospital for Children, London, UK

## Background

Severe degenerative calcific aortic stenosis (cAS) affects 3% of the over 75 year-olds and leads to heart failure and death unless the valve is replaced. Coexisting cardiac amyloid - typically wild-type Transthyretin (wtATTR) has been reported but has not been systematically studied: its prevalence and prognostic significance are unknown. Multiparametric CMR (function, LGE, T1 and ECV mapping) has high sensitivity and specificity for cardiac amyloidosis. We sought, in cAS, to define the prevalence of occult cardiac amyloid by biopsy, subtype it, explore its CMR presentation and understand its prognostic significance.

## Methods

As part of the RELIEF-AS study (NCT 02174471), 146 patients with severe AS awaiting aortic valve replacement (AVR) underwent CMR (LGE, T1 mapping) and intra-operative myocardial biopsies. 108 patients had calcific AS (cAS) [75 ± 6 years; 58% male]; the remainder had bicuspid (36), rheumatic (1) or unicuspid AS (1). Biopsies were screened for cardiac amyloid by congo red staining; if positive, tissue was fully sub-typed (immunohistochemistry, mass spectrometry as needed), and patients underwent full clinical amyloid assessment including genotyping and DPD (bone tracer) scintigraphy.

## Results

**Figure 1:**
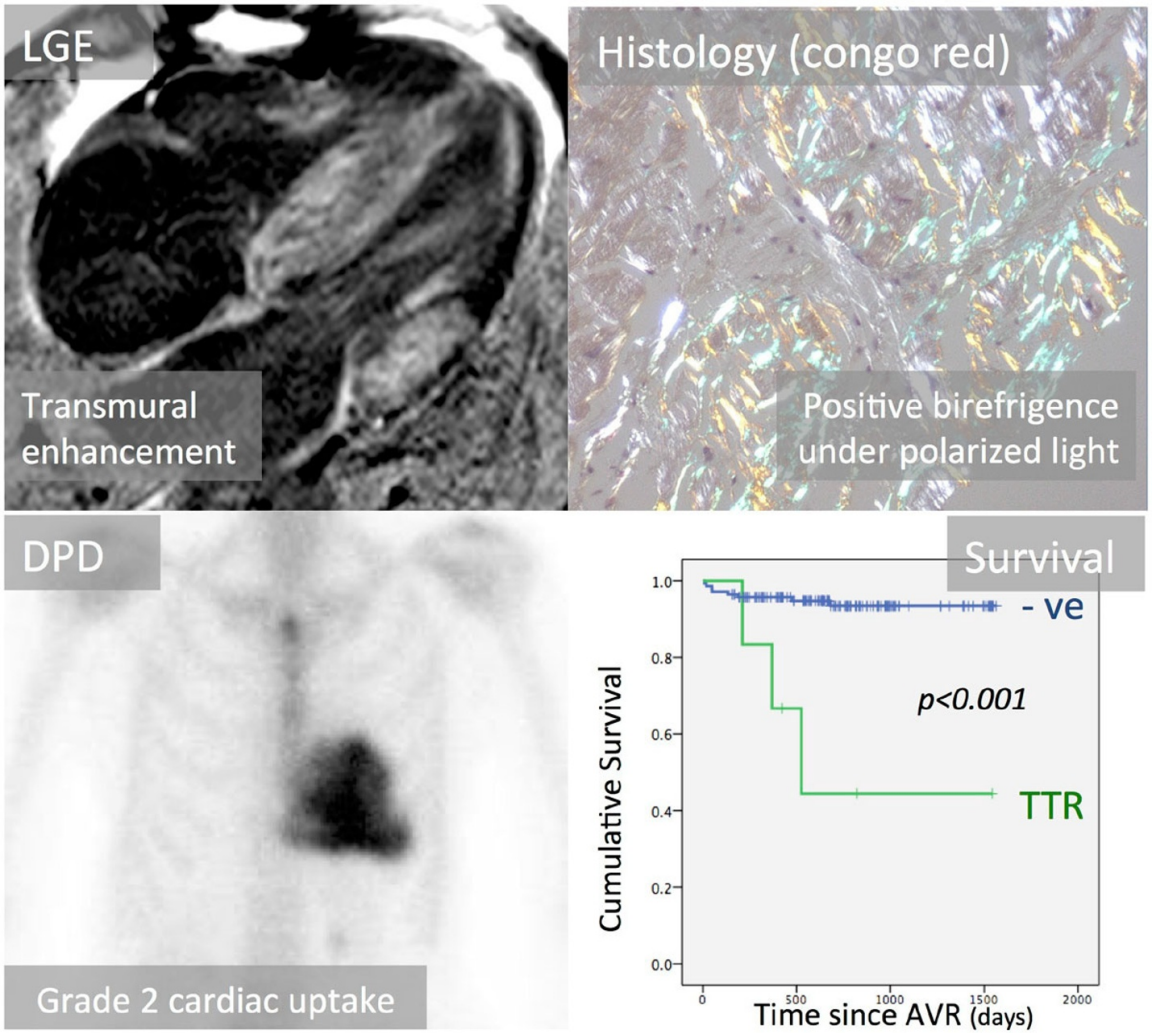
**Occult Transthyretin Amyloid Deposition in Aortic Stenosis; detected by CMR late gadolinium enhancement (LGE), histological congo red staining and DPD scintigraphy; Transthyretin deposits are associated with poor outcome in aortic stenosis (Kaplan-Meier-Plot)**.

Myocardial biopsy identified amyloid depositions in 6 out of 108 cAS patients (all age >65 years); none in the other cohorts. All were TTR. All were by genotyping wild-type. Two had definite cardiac amyloidosis by CMR and DPD, but were not recognized by routine pre-operative work-up (Figure 1). Four other patients had findings by multiparametric CMR that could be explained solely by severe AS (Table 1). At median follow-up of 1.9 years (0.4-4.2 years), 50% of the affected patients had died compared to 7.8% in the remaining cAS cohort. This was highly significant and, of all parameters assessed, the presence of TTR amyloid had the highest hazard ratio for death (HR 9.4 [2.4-35.6], *p = 0.001,* univariable Cox regression analysis).

**Table 1:** Pre-operative characteristics of patients with TTR on cardiac biopsy

	Patient 1	Patient 2	Patient 3	Patient 4	Patient 5	Patient 6
**Age/Gender**	73 female	69 male	80 female	85 male	84 male	71 male
**Biopsy/Genotype**	TTR/wild-type	TTR/wild-type	TTR/wild-type	TTR/wild-type	TTR/wild-type	TTR/wild-type
CMR LGE pattern*	Amyloidosis	Amyloidosis	AS	AS	AS	AS
**ECV**	60%	52%	31%	25%	32%	32%
**LV mass index (g/m** ^**2**^ **)**	137	150	117	101	93	132
**DPD Scintigraphy**	Grade 2	Grade 2	NA	Grade 1	NA	Grade 1
**Peak AV Gradient (mmHg)**	74	45	70	110	61	116
**AVAi (cm** ^**2**^ **/m** ^**2**^ **)**	0.36	0.52	0.6	0.34	0.35	0.24
**Follow-up Status**	Alive	Dead	Dead	Alive	Dead	Alive

*LGE pattern consistent with diagnosis of cardiac amyloidosis / aortic stenosis (AS)

## Conclusions

Occult cardiac amyloid (wild-type TTR) has a prevalence of 6% in cAS undergoing surgical AVR (mean age 75). It has a poor outcome even when at low levels of infiltration. Multiparametric CMR becomes diagnostic in 1/3, but early disease looks like the changes of AS - here, DPD scanning adds value.

